# Network resilience of the human interactome to pan-cancer mutations reveals conserved pathway vulnerabilities

**DOI:** 10.1016/j.isci.2026.115500

**Published:** 2026-03-27

**Authors:** Stefano Polizzi, Nicolas Biondini, Tommaso Matteuzzi, Martina Tarozzi, Gastone Castellani

**Affiliations:** 1 IRCCS Istituto delle Scienze Neurologiche di Bologna, Data Science and Bioinformatics Laboratory, 40139 Bologna, Italy; 2 Department of Medical and Surgical Sciences (DIMEC), University of Bologna, Bologna, Italy; 3 Department of Physics and Astronomy, University of Firenze, Firenze, Italy; 4 IRCCS Azienda Ospedaliero-Universitaria di Bologna S.Orsola, 40138 Bologna, Italy

**Keywords:** cancer systems biology, cancer

## Abstract

Interactomes encode interdependencies among molecular components in the cell. Gene mutations are seen as node failures that affect the biological information flow and possibly lead to diseases. Using an information-based resilience measure, we study how the overall topology of the human interactome is affected by tumor-related (pan-cancer) mutations and linked to functionality, aiming to reveal common mechanisms. We found that cancer-associated mutations disrupt the interactome significantly more than random failures, leading to faster network fragmentation. The impact of the single gene on the resilience shows two well-separated groups. Most mutations are consistent with random occurrence, but a small subset (664), undetectable through standard metrics, plays a pivotal role in maintaining network integrity and, consequently, cellular functionality. This allows the identification and ranking of crucial genes. Enrichment analysis shows the involvement of those genes in apoptotic and other relevant biological processes that are not enriched in the larger group and are conserved in cancers, supporting the concept of cancer as a disease emerging from system-level dysregulation.

## Highlights

• Pan-cancer mutations disrupt human interactome resilience more than random failures
• *Local hypothesis* is significant and is due to higher clustering of mutated genes
• A small subset of high-impact ranked genes preferentially affects network integrity
• Apoptosis pathways are enriched among high-impact mutated genes

## Graphical abstract

**Figure:**
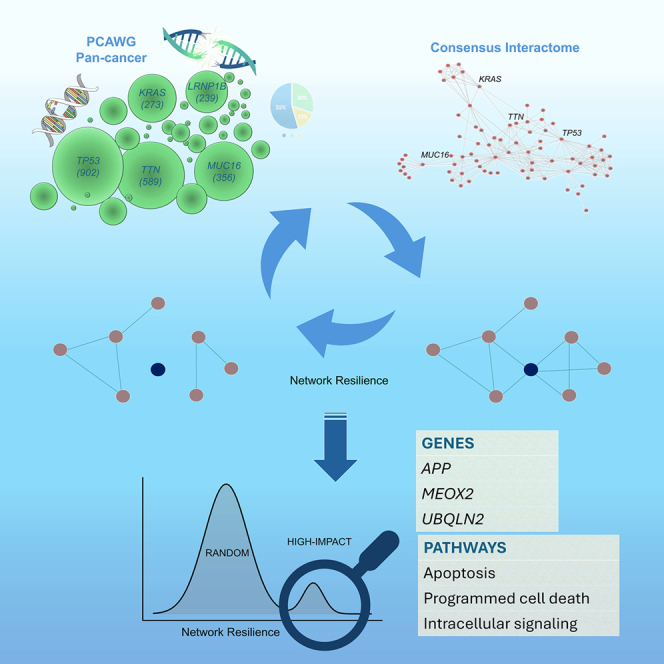

## Introduction

A major focus of recent oncological research has been the identification of common patterns in all types of tumors.^1^^,^^2^^,^^3^^,^^4^^,^^5^ The onset and progression of cancer are generally attributed to the accumulation of genetic alterations, a principle underlying the concept of driver genes. Driver genes, often shared among several cancers, have improved cancer treatment, yet they do not fully explain cancer onset or heterogeneity. Despite advances in targeted genetic therapies, several cancers exhibit high relapse rates, and such treatments often show limited efficacy.^6^ In addition, a substantial proportion of patients lack identifiable driver mutations.^7^ Although cancer presents tissue-specific features, shared molecular mechanisms have been discovered and are critical for prevention and diagnosis. This means that models can identify shared prognostic information that is applicable beyond a single histological tumor type and then improve cancer understanding and treatment.^1^^,^^5^ Interestingly, we now understand that cancer cells acquire selective growth advantages not only through genetic mutations, but also through functional alterations in post-transcriptional regulation, such as RNA splicing.^8^ Probably, the most outstanding example of common mechanisms is the Warburg effect,^9^ which acts on the metabolic pathway of cancer cells, but other mechanisms have been investigated, and the importance of the microenvironment, the cell niche, and even epigenetics has recently been brought to the forefront by several studies.^5^^,^^10^^,^^11^ Indeed, experiments show that numerous precancerous lesions carrying known oncogenic mutations (such as *KRAS*) do not always lead to tumor formation,^11^ confirming that somatic genetic mutations are not the full explanation for cancer.^12^^,^^13^ In addition, rare events in certain cancer subgroups may be common enough in a pan-cancer dataset for models to effectively assess their prognostic impact. In our study, we analyze the effect of pan-cancer genetic mutations, including brain cancer, on the resilience of the interactome network, leveraging the whole set of mutations that allows one to also reveal the effect of mutations that would be very rare in a single cancer type.

A possible solution to the unresolved question that somatic mutations are not the full explanation is to consider that cancer is not the result of alterations in one or a few specific genes. It should be considered more as a perturbation of the cellular state arising from dysfunctional molecular interactions.^12^ The same cancer can originate from completely different sets of mutations, and a wide heterogeneity of mutational landscapes is observed between patients and cancer types.^14^^,^^15^ This variability hampers the efforts to link cancer genotype to phenotype. It is thus crucial to uncover interdependencies among gene alterations, i.e., how they “collaborate” to give rise to cancer. A first step in this direction is to study the regularities of the location of disease genes on biological networks, such as their proximity to hubs or their tendency to form disease modules.^16^^,^^17^^,^^18^ This tendency for disease-related genes to have increased interactions with each other, known as the *local hypothesis*, is an often-unverified assumption in network medicine and has as a corollary that genes involved in cancer should have a larger clustering coefficient than non-mutated genes. We evaluated this effect in the pan-cancer pathology to test the assumption that multiple cancer types display overlapping phenotypic characteristics.

For this reason, this study leverages the architecture of the network of interactions between genes to characterize genes' position and function. Recent advances in experimental techniques^19^^,^^20^ to uncover interactions between cell constituents have made available several different maps of biological interactions; among them, the most studied is the interactome or protein-protein interaction (PPI) network. The human interactome is a network where each node represents a gene and an edge represents an interaction between two genes or their products. In this context, a mutation can be seen as the removal of all interactions of a node (e.g., a misfolded protein). In other words, a mutation is a network failure that affects the flow of biological information in the cell network.^21^^,^^22^

**Figure 1:**
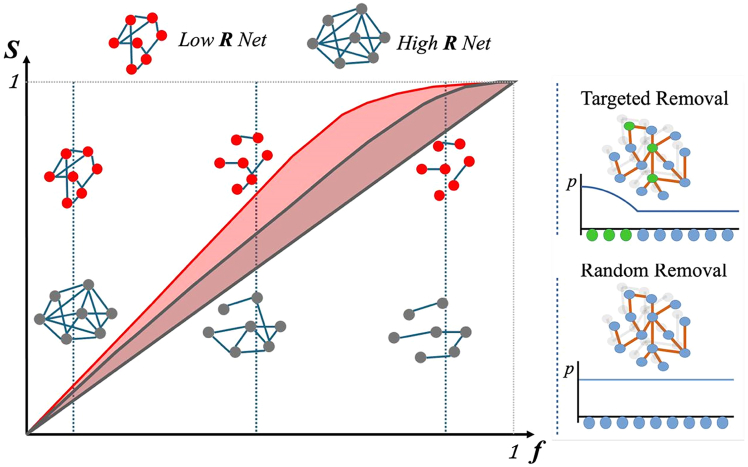
Schematic drawing of the resilience concept R and removal procedure Illustrative example of the Shannon diversity *S* as a function of the fraction of removed nodes *f*. The higher the network fragmentation the higher *S*. *R* is lower when the integral of *S* in [0,1] is greater. Right: Node removal schemes. In random removal, nodes are chosen with a uniform probability, while in targeted removal, ’target’ nodes are removed with a predefined order.

Resilience, or fault tolerance, quantifies the extent to which a network changes when one or more nodes (or links) are removed. The lower the change, the higher the resilience (Figure 1). The more fragmented the network is because of the failure of one gene, the more vital that gene may be for maintaining cellular function. Resilience of the interactome is a critical property, the lack of which can lead to cell death or disease, e.g., cancers. In the context of biological networks, several resilience measures have recently been proposed. Here, we chose the resilience presented by Zitnik et al.,^22^ which is a global measure derived from Shannon diversity of the fragmentation of the network into separated sub-networks, integrated over all possible failure rates, from 0 (no node is removed) to 1 (all interactions are removed).

Here, we show that the human interactome exhibits resilience properties distinct from standard network models, consistent with evolutionary optimization. By integrating pan-cancer mutations from the extensive and complete database of Pan-Cancer Analysis of Whole Genomes (PCAWG)^2^ with this network framework, we quantify how somatic mutations affect interactome integrity, identify genes with disproportionate impact on resilience, and benchmark these effects against random and hub-targeted failures. All results are validated on the TCGA pan-cancer database.

## Results

Networks are an efficient way to characterize the overall architecture of interactions among cellular molecular constituents, giving a picture of their global interdependencies. Studying the extent to which the topology of a biological network is affected by failures, i.e., the removal of nodes, enables us to advance hypotheses on the loss of biological functions and the increased risk of diseases.

### Comparison of interactome resilience with standard networks

Biologically important genes in the interactome are usually genes that have a predominant position in the network; therefore, besides considering random failures, here we analyze targeted failures of nodes in decreasing number of connections, and study the resilience of the interactome in comparison with common standard network topologies. We compared interactome resilience with respect to random regular networks (RNs), Erdős-Rényi (ER), scale-free (SF), and fully connected (FC) networks. Since *S*(*f*) depends on link density, we compared each reconstruction to the above network models with the same density as the interactome.

Under random genetic mutations, the interactome is slightly less resilient than the corresponding SF network (Table 1). However, all resilience values are close together, which means that the interactome is globally well resilient to random mutations. Interestingly, under targeted removal, there is instead a drop in the resilience of both SF and interactome networks. This suggests that evolution optimized the interactome to resist random mutations, which are more frequent in normal conditions, but made it vulnerable to highly connected nodes, similarly to the behavior of the SF network.

**Table 1:** Resilience values

Network	Random Removal Resilience (sem)	Targeted Removal Resilience (sem)
Fully Connected	0.473(1.1 × 10^−17^)	0.473(1.1 × 10^−17^)
Random Regular	0.474(5 × 10^−07^)	0.470(1.5 × 10^−06^)
Erdős–Rényi	0.472(5.5×10^07^)	0.469(1.7 × 10^−06^)
Scale-Free	0.463(2 × 10^−05^)	0.305(3 × 10^−05^)
Interactome	0.458(4 × 10^−5^)	0.301(9 × 10^−06^)

Resilience mean values (standard error of the mean) of common standard networks and the interactome.

**Figure 2:**
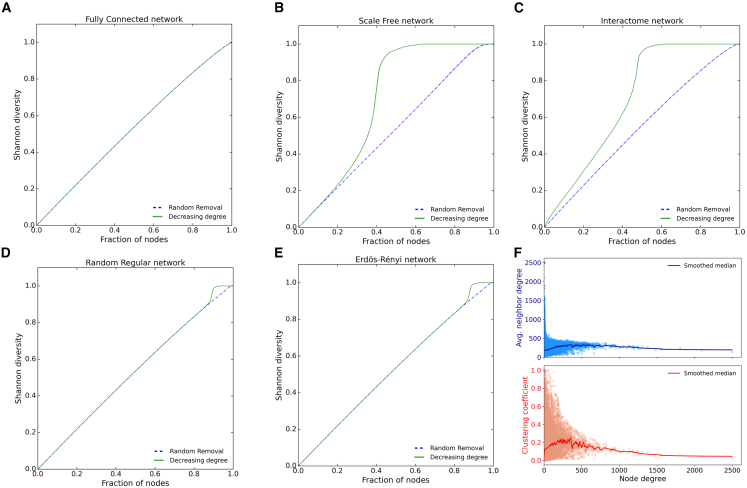
Shannon diversity curves for standard networks Shannon diversity curves and standard error intervals (shaded areas) for random and decreasing-degree failure, comparing the interactome (C) with standard network structures with the same link density and the same number of nodes as the interactome: fully connected (A), scale free (B), random regular (D), and Erdös-Rényi (E). Note that in all previous plots the maximum possible resilience is given by the bisectrix. (F) Scatterplot of the clustering coefficient (cc in red) and of the average degree of the first neighbors (blue), of the consensus interactome as a function of the node degree. Dots are nodes, and solid lines are Gaussian-smoothed medians.

Figure 2 shows the typical trend of *S*(*f*) for the interactome and the related network models. Fixed *f*, *S*(*f*) is minimum when only one connected component is present, and *S*(*f*) would be the diagonal for *N*→*∞* (see Methods S1). The FC network has the highest resilience and is the closest to the bisectrix, the difference being due to a finite-size effect. The RR and ER networks also have a high resilience to node removal, but show little effect due to the random structure that creates more vulnerable nodes, even though the effect appears only after 80% of the network has already been disconnected. In fact, this is negligible for practical purposes, since when more than 80% of the network is disrupted, the network structure has already disappeared. The SF and interactome networks instead have a notably different behavior, especially under targeted removal. Indeed, the plots show two very different classes of network resilience: FC, random regular, and Erdős-Rényi networks with similar resilience behavior, while the interactome behaves similarly to a SF network, even though the degree distribution is not SF (see Figure S1). Its resilience is more susceptible to targeted attacks than that of a SF network, as shown in Figure 2B, where the two curves begin to diverge from the outset—after the removal of only a small fraction of hubs—whereas in the SF network this occurs only after approximately 20% of nodes are removed (Figure 2). This indicates that the network begins to lose functionality under relatively small targeted perturbations, earlier than the other networks, revealing greater brittleness. Consequently, its organization appears to be even more crucial than in a SF network, with gene interactions exhibiting an evolutionarily shaped structure.

Figure 2F shows some properties the interactome: as an SF network most of the nodes have a low degree and only a small fraction are hubs, but the degree distribution (shown in Figure S1) is not a straight line as it is for SF networks; instead, it is better fitted by a log-normal tailed distribution (*p* value = 0.03 of the comparison test^23^). This is coherent with many observed degree distributions and biological processes, even in cancers.^24^^,^^25^ The average neighbor’s degree (blue) shows a slightly disassortative network organization, where hubs tend to connect preferentially to low-degree nodes. Figure 2F also shows the clustering coefficient (cc, in red) for all nodes: nodes with small degrees tend to be connected to each other more than nodes with high degrees. This is different from what is observed with typical SF networks, where the clustering coefficient decreases exponentially with the system size, leading to values around ∼10^−3^ for a comparable number of nodes,^26^ and reflects the modular and hierarchical organization of the interactome that likely leads to its peculiar behavior.

### Interactome resilience to pan-cancer mutations

Besides investigating the resilience of consensus interactome to random and targeted gene failures, we are interested in the impact of pan-cancer associated genes on the network topology and aimed to quantitatively evaluate the effect of the “local hypothesis”. To this end, we simulated gene failures in decreasing order of frequency of mutations for the pan-cancer pathology. If cancer mutations are not randomly distributed within the interactome, one would expect a difference in global resilience with respect to randomly selected mutations. For each gene, we counted the number of samples with one or more mutations and removed genes in descending order (i.e., decreasing mutation frequency), randomly sampling among genes with identical counts.

**Figure 3:**
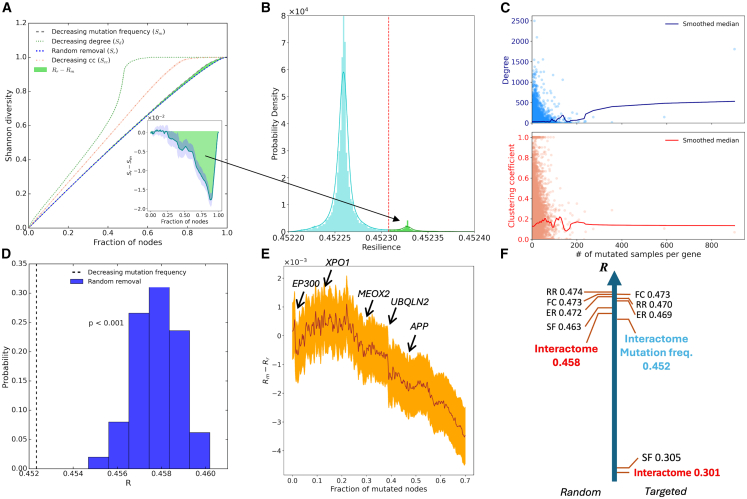
Results about pan-cancer mutations (A) Shannon diversity curves of the interactome with different removal types, in order of decreasing mutation frequency (gray) and decreasing clustering coefficient (salmon) in comparison with previously shown curves. Lines are the means, and the shaded areas are the standard error intervals. Inset: difference of the Shannon diversity between random removal and decreasing frequency of mutation. (B) Probability density of resilience values after every single mutated gene is randomly considered as not mutated: higher resilience values mean higher gene impact. (C) Scatterplot of the clustering coefficient (cc in red) and of the node degree, of the consensus interactome as a function of the number of samples with one or more mutations per gene. Dots are nodes, and solid lines are Gaussian-smoothed medians. (D) Distribution of resilience values for random removal and value of resilience for decreasing mutation frequency (dotted line). *p* value of the *t* test. (E) Mean (solid line) and standard error interval (shaded area) of *R*_*m*_-*R*_*r*_, respectively, resilience under gene removal in decreasing order of mutation frequency and resilience under random removal, when cumulatively removing mutated genes (grouped by five). (F) Ordered summary of resilience values for standard networks and consensus interactome, when removing genes randomly (left) or in decreasing degree/mutation frequency (in blue) order (right).

We observed a lower resilience to cancer mutations, which is significantly lower than for random mutations (Figures 3A and 3D), respectively *R*_*m*_ = 0.452365 ± 9×10^−6^ and *R*_*r*_ = 0.45779 ± 4×10^−5^, where the error is given by the standard error on the mean. The *p*-value of the *t* test is < 0.001, the absolute difference is *R*_*r*_-*R*_*m*_ = 0.00542 ± 0.00005, and 95% confidence intervals are *R*_*m*_ = [0.452347,0.452384] and *R*_*r*_ = [0.45770,0.45787]. Computed on 500 repetitions for *R*_*r*_ and 100 repetitions for *R*_*m,*_ which does not contribute to the global error, since the order is almost deterministic. The global effect on resilience is relatively small when compared to targeted removal, and the shape of the Shannon diversity curve is very similar to the random one, compared to the degree of order. In fact, this result is in line with the “local hypothesis” which states that mutated genes involved in the same disease tend to be neighbors on the interactome, and therefore to disrupt the network faster. Indeed, in this way, it is more likely to isolate connected regions, making the network more unstable. The inset in Figure 3A shows a different visualization of the same result, but in the form of the curve difference, *S*_*r*_-*S*_*m*_. The shaded area is the standard error given by random repetitions. The drop in resilience is more remarkable here than in the Shannon diversity curves and occurs around a fraction of node failure of 0.6. We can then conclude that somatic mutations in cancer preferentially affect the interactome in a manner that is not entirely random, though less dramatically than targeted hub removal. These results are identical in the validation data (Figure S2).

We investigated in more depth the reasons for this drop and tried to understand the meaning of the difference between mutation-based and random removal, which could hide some biologically important reasons, and some common patterns of the pan-cancer pathology. To better understand the reasons for the particular trend of the mutational curve we first removed genes with the same degree as the genes removed in order of frequency of mutations, but selected randomly, and the observed difference was no longer there, suggesting that mutated genes are selected biologically, and their degree is not the cause of the observed difference (see Figure S3). Therefore, pan-cancer mutations generally do not occur in genes with specific degrees, but rather at a specific position. We then asked whether certain genes occupy particularly central positions whose inactivation could drive network fragmentation and sought to identify them. The Shannon diversity metric captures both the individual contribution of each gene and the cumulative (or memory) effect of all previously inactivated genes. Thus, the observed drop around *f* = 0.6 is not necessarily and not only due to genes selected around that value, which are, however, the first promising candidates, but may also be a nonlinear threshold effect cumulated from high-frequency mutated genes.

Then, we studied the effect of a single gene perturbation on the whole biological information flow and measured its impact on system-level robustness. For each gene, we recomputed the full resilience analysis after excluding that gene from the list of mutated genes, without altering the mutation-frequency ordering of the remaining genes. The excluded gene was therefore treated as non-mutated and removed only as part of the non-mutated background. This procedure captures the incremental contribution of each gene to the global resilience curve, while preserving the cumulative (memory) effect of all genes removed before it. The results shown in Figure 3B reveal two distinct sets of genes. We separated the two sets by setting a cutoff on the right side of the higher peak in such a way that it exhibits left-right symmetry. A small group of genes (664) is well separated and exerts a stronger impact on interactome resilience. In fact, the distribution of Figure 3B shows that if those genes were not mutated, the interactome would be more resilient (*R* would be higher); therefore, they are mainly responsible for the observed increased fragmentation of cancer-mutated genes. This effect cannot be attributed solely to the structural properties of the interactome or to the degree of the mutated genes. When the same analysis was performed by replacing the list of mutated genes with genes ordered by decreasing degree (excluding one gene at random from the list), the resulting distribution displayed only a single main peak, and the two distinct gene populations were no longer observed (Figure S4), showing that the two peaks are not characteristic of the topology of the interactome alone, but instead due to the specificity of mutated genes. In Figure 3E, we analyzed the previously cited memory effect. We plotted the difference *R*_*m*_-*R*_*r*_ and its standard error obtained by subsequently adding groups of five genes in decreasing order of mutation frequency to the list of genes to be removed. Specifically, we first removed the five most frequently mutated genes, then the top ten, fifteen, and so on, progressively including less frequently mutated genes while randomly removing the remaining ones. In this way, we can verify whether and how many of the most frequently mutated genes have an impact on the overall resilience. We conclude that the top 25% of the most mutated genes have no effect on the genes that are removed afterward. Nonetheless, a memory effect is observed: Resilience begins to decline linearly around 25%, with the largest drop occurring just before the top 40% of mutated genes. We can therefore say, for instance, that at *f* = 0.4, around 1/5 of the resilience difference is explained by previously removed genes, and the remaining by genes removed later. Interestingly, after *f* = 0.25 the effect on network resilience is irreversible and, even if subsequent genes are removed randomly, it remains there.

The set of high-impact genes was compared to the COSMIC v103 gene catalog,^27^ after filtering for the Cancer Gene Census (CGC), which contains 590 high-confidence cancer drivers. Of these known cancer driver genes, only 76 are in common with the list of 664 high-impact genes we obtained. This suggests that while some of the highest-impact genes correspond to well-established cancer drivers, the majority of the resilience-critical genes identified here are not annotated as drivers in COSMIC, indicating that our approach captures network-level vulnerabilities beyond canonical oncogenes.

In summary, we are able to identify genes due to the network resilience measure that we would not be able to identify otherwise. Table 2 shows the genes with the greatest impact on resilience and compares them with the nodes with the highest degree. Only 4 of them can be considered hubs, while the others have a position inside the network that causes a high network fragmentation and are necessary to keep the cell alive. Moreover, the genes with the greatest impact are not those most frequently mutated.

**Table 2:** High-impact genes

Highest Degree				Most impactful on Resilience			
Gene	Degree	# Mut Samp	Resilience	Gene	Degree	# Mut Samp	Resilience
*APP*	2497	11	0.453769	*APP*	2497	11	0.453769
*NTRK1*	2096	22	0.452373	*MEOX2*	663	16	0.453246
*TP53*	1807	902	0.452449	*UBQLN2*	362	14	0.452790
*EGFR*	1555	49	0.452485	*EP300*	1253	53	0.452574
*GRB2*	1546	10	0.452487	*XPO1*	1483	28	0.452568
*XPO1*	1483	28	0.452568	*SRPK1*	479	15	0.452552
*HSP90AA1*	1375	12	0.452359	*SRPK2*	488	18	0.452546
*ESR1*	1308	19	0.452359	*MCM2*	1099	16	0.452539
*YWHAB*	1280	6	0.452312	*UBQLN1*	345	15	0.452496
*EP300*	1253	53	0.452574	*GMCL1*	134	11	0.452496

Comparison between the 10 nodes with the highest degree and the 10 nodes with the highest resilience impact, measured as in Figure 3B, when the gene is considered as non-mutated. The number of samples in which that gene was found mutated is # Mut Samp. The full list of genes is in Table S2.

Subsequently, we removed each gene and its first neighbors to evaluate in more detail the “local hypothesis”. In this case, resilience drops to 0.379 for random removal and to 0.388 for mutation-based removal (see Figure S5 and Table S1). This case represents a direct measure of the “local hypothesis”. The fact that the resilience for mutation-based removal is here higher than that for random removal, differently from single-node results, strongly suggests that mutated genes tend to be clustered together, providing a protective effect against fragmentation, and it is likely an effect of the significantly higher clustering coefficient of mutated genes (Wilcoxon-Mann-Whitney test *p*-value <0.001, Figure S6; Figure 3C). This result has a biological root: Often genes that are in the same interactome neighborhood (or module) share the same functions, participate in the same protein complexes, or are involved in the same processes. Therefore, protein complexes involving cancer-mutated genes compensate for mutation-induced impairments by being more tightly interconnected.

Figure 3F shows a summary of all the main resilience measures analyzed in this study. The total variability is in the range [0.3,0.46], so it is not very large compared to the whole range of possible resilience. The maximum possible resilience for a network with the same property as the interactome is 0.475, given by the formula in Methods *S1*, while high-degree nodes are the most important for network integrity.

### Functional enrichment of vulnerable nodes

In this section, we analyze and compare the two groups of genes identified in the previous section (Figure 3B). We performed functional enrichment with over-representation methods on the Gene Ontology (GO) database and studied the processes that are statistically overrepresented in the two groups of genes with respect to the whole human genome.^28^^,^^29^ The results (Tables 3 and 4) show that the set of genes causing the greatest decrease in interactome resilience is significantly enriched in apoptotic processes, including peptidyl-tyrosine modification, as peptidyl-tyrosine residues can affect proteins involved in programmed cell death,^30^ influencing cell survival. On the other hand, the genes corresponding to the main peak of the distribution in Figure 3 exhibit enrichment in broader nonspecific processes, distinct from those associated with the smaller group, such as transcriptional regulation and ion transport. Together with previous results (Figure 3A and 3B; Table 2), this confirms the hypothesis that cancer mutations appear mainly randomly, but a small subset of them causes a decrease in interactome resilience. If all mutated genes impaired interactome resilience, the cell would undergo functional failure, precluding any evolutionary advantage. A similar result was also found using the TCGA database. In particular, the first 68 processes with the highest adjusted *p* value of the PCAWG are also relevant processes (adjusted *p* value < 0.05) for the set of high-impact genes identified in the same way in the TCGA database. Moreover, 90 genes out of the 100 genes with the greatest impact on resilience in the TCGA are also in the described set of high-impact genes.

**Table 3:** Enrichment analysis (high-impact genes)

GO Term	Adjusted P-value	Overlap
Regulation of Apoptotic Process (GO:0042981)	3.54 × 10^−10^	67/704
Negative Regulation of Apoptotic Process (GO:0043066)	2.44 × 10^−8^	49/475
Negative Regulation of Programmed Cell Death (GO:0043069)	6.52 × 10^−8^	42/382
Intracellular Signaling Cassette (GO:0141124)	1.72 × 10^−7^	39/354
Peptidyl-Tyrosine Modification (GO:0018212)	1.72 × 10^−7^	14/46
Positive Regulation of Cellular Process (GO:0048522)	1.72 × 10^−7^	55/622
Positive Regulation of Intracellular Signal Transduction (GO:1902533)	2.99 × 10^−7^	56/652
Cell Surface Receptor Protein Tyrosine Kinase Signaling Pathway (GO:0007169)	1.99 × 10^−6^	33/296
Regulation of Cell Population Proliferation (GO:0042127)	2.20 × 10^−6^	60/769
Protein Modification by Small Protein Conjugation (GO:0032446)	2.32 × 10^−6^	34/316

Top 10 GO biological processes enriched in the set of genes with the highest resilience impact on the network. The full list of enriched processes is in Table S3.

**Table 4:** Enrichment analysis (low-impact genes)

GO Term	Adjusted P-value	Overlap
Regulation of DNA-templated Transcription (GO:0006355)	4.07 × 10^−18^	1937/2139
Regulation of Transcription by RNA Polymerase II (GO:0006357)	4.91 × 10^−16^	2025/2250
Positive Regulation of DNA-templated Transcription (GO:0045893)	1.78 × 10^−9^	1152/1274
Positive Regulation of Transcription by RNA Polymerase II (GO:0045944)	1.06 × 10^−6^	888/983
Monoatomic Cation Transmembrane Transport (GO:0098655)	3.31 × 10^−6^	288/304
Inorganic Cation Transmembrane Transport (GO:0098662)	6.46 × 10^−5^	288/307
Nervous System Development (GO:0007399)	1.61 × 10^−4^	440/480
Potassium Ion Transmembrane Transport (GO:0071805)	6.59 × 10^−4^	130/134
Potassium Ion Transport (GO:0006813)	1.44 × 10^−3^	123/127
Positive Regulation of RNA Biosynthetic Process (GO:1902680)	1.44 × 10^−3^	502/555

Top 10 GO biological processes enriched in the set of genes from the main peak (low-impact) of the distribution in Figure 3B.

It is interesting to note that genes belonging to the high-impact set are mostly enriched in both processes: regulation of apoptosis and inhibition of apoptosis. Both processes interestingly cause irreversible damage to the interactome network, which means that the resilience of the interactome is crucial to maintain cell functionality, and the apoptosis mechanisms are key for that. This correspondence between network integrity and cell functionality emerged through the resilience-based ranking.

**Figure 4:**
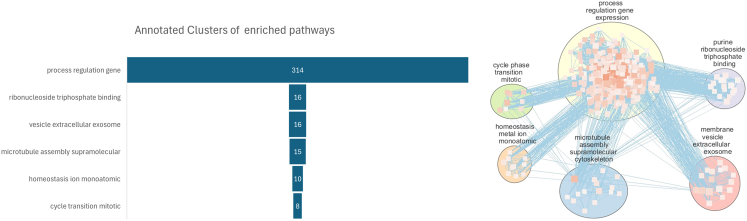
Grouped functional modules Representation of the enriched pathways grouped by non-redundant functional modules.

Among the most impactful nodes identified based on their degree centrality and influence on global network resilience, we found a convergence on a set of 17 genes, either of which removal disproportionately disrupted global interactome connectivity or whose degree centrality within the interactome is extremely high. These include both well-established oncogenes and tumor suppressors such as tumor protein P53 (*TP53*), epidermal growth factor receptor (*EGFR*), and neurotrophic receptor tyrosine kinase 1 (*NTRK1*) which emerged as critical hubs, consistent with their known roles in regulating cell cycle, proliferation, and survival.^31^ The presence of estrogen receptor 1 (*ESR1*) and E1A binding protein P300 (*EP300*) further underscores the importance of transcriptional regulation and hormone signaling in maintaining network cohesion.^32^ This is also confirmed by the second most impactful gene on network resilience, mesenchyme homeobox 2 (*MEOX2*), a gene upregulated in glioblastoma,^33^ that encodes a transcription factor that influences cell-cycle progression and survival and whose mechanistic role in cancer is not yet well understood, although its influence on cell-cycle progression and survival is consistent with its high impact on network integrity. Signal transduction mediators such as growth factor receptor-bound protein 2 (*GRB2*), *YWHAB* (14-3-3 Beta), and exportin 1 (*XPO1*) were also prominent, reflecting their centrality in oncogenic signaling cascades.^34^^,^^35^ amyeloid precursor protein (*APP*), which results first in both rankings, is important for neural transmission and highly expressed in brain,^36^ it is then not surprising that it is overexpressed in glioblastoma, but it is also interestingly mutated in multiple cancers, such as breast, pancreatic, lung, colon, and prostate.^37^ Furthermore, heat shock protein 90 alpha family class a member 1 (*HSP90AA1*), and ubiquilin 1/2 (*UBQLN1*/*UBQLN2*) highlight the role of proteostasis in network stability, suggesting that alterations in protein folding and degradation can propagate destabilizing effects throughout the network.^38^^,^^39^^,^^40^ Beyond classical drivers, several high-impact genes converge on processes highlighted by our GO enrichment. *MCM2*, a replication-licensing factor, connects resilience sensitivity to replication stress, and *SRPK1/2* further support the involvement of splicing regulation as a network-level vulnerability. Together, these mechanistic roles illustrate how resilience prioritization identifies genes whose functional perturbation is tightly coupled to apoptotic and transcriptional integrity. The importance of splicing is consistent with what was found in recent pan-cancer studies, as previously mentioned.^8^ Moreover, these genes are already known to be involved in oncogenesis and chemotherapy sensitivity and resistance.^41^^,^^42^^,^^43^ Their network criticality highlights that even genes with moderate connectivity can exert disproportionate effects when positioned at central or bridging points in functional subnetworks. In particular, only a minority of these vulnerability-associated genes were hubs in the traditional topological sense. This indicates that degree centrality alone is insufficient to predict the functional consequences of gene loss, and that resilience-based metrics offer complementary insights. Figure 4 represents the enriched processes grouped by non-redundant functional modules. Most biological processes can be grouped into the functional module of gene expression regulation.

## Discussion

We found that the PPI network has a similar resilience behavior to a scale-free network, but has a higher modular structure, which makes it more vulnerable to targeted perturbations, revealing greater brittleness. This can be explained by the gene interactions exhibiting an evolutionarily shaped structure; in fact, biological modules participate in the same protein complexes or are involved in the same pathways. When considering the mutation frequency of pan-cancer genes, we found a very interesting bimodal distribution, identifying a small portion of genes with a greater impact on resilience. Those genes have specific topological properties that make them essential to preserve the network integrity and cannot be identified by simple metrics, such as degree or frequency of mutations.

The genes identified with our analyses confirm the importance of transcriptional regulation and splicing in cancer processes, such as *MEOX2*, *EP300*, *SRPK1*, *SRPK2,* and *MCM2*. Genes that preserve network integrity despite not being highly connected are likely involved in cancer-related processes that are often overlooked by current therapeutic strategies, yet our results show they play a crucial role. Functional enrichment analysis revealed that genes with a high impact on interactome resilience are significantly enriched in the apoptotic signaling and intracellular signaling processes. These processes are essential for cellular stress surveillance and fail-safe mechanisms, and their compromise could lead to increased cellular heterogeneity and malignant transformation. Interestingly, this enrichment was not observed in the main set of frequently mutated genes, which instead mapped to more generic biological processes, such as transcriptional regulation and ion transport. This supports the interpretation that, while many cancer mutations are functionally neutral or passengers, a small but consistent fraction targets genes critical for preserving the robustness of cellular interaction networks. This finding is coherent and well supported by previous pan-cancer studies.^44^^,^^45^ The fact that high-impact genes alter apoptotic processes is explainable by the fact that cancer cells are not eliminated by the immune system, but this is done in a way that does not completely disrupt the network, consistent with the fact that otherwise the cell would cease to function completely.

Taken together, our findings emphasize that the human interactome has evolved to buffer against random genetic perturbations, but remains vulnerable to mutations targeting structurally and functionally strategic nodes. These network-centric vulnerabilities are not fully captured by traditional metrics such as the number of mutated samples per gene or node centrality, underscoring the value of resilience-based approaches for uncovering latent structural weaknesses. The discovery and identification that certain weak points (genes/complexes/processes) are consistent across all cancer types, especially in apoptosis and intracellular signaling processes, offers potential for the development of therapeutic strategies aimed at reinforcing or re-routing network connectivity in the face of oncogenic stress. Resilience should be considered in future studies to identify key genes in cancer that could provide new insights into ongoing processes and potentially be drug targets: Targets selected from high-impact genes have a higher probability of application in several types of cancer. This has immediate applications for experimental validation and translational development; Perturbation experiments targeting high-impact genes, alone or in combination, could test their predicted role in buffering network integrity. For instance, by CRISPR knockout/CRISPRi of high-impact genes together with a control group of genes, cell viability metrics and apoptosis sensitivity can be measured and compared. At the translational level, these genes may represent novel vulnerability markers, whose targeting could sensitize cancer cells to stress or conventional therapies by increasing interactome resilience (restoring healthy resilience) rather than, or in conjunction with, directly acting on driver genes. This can be tested by differential expression analysis targeting high-impact genes in cancer cell lines and untreated controls.

The aim of this study is to identify and rank genes according to their impact on the exchange of any biological information between proteins in a cell. Genetic mutations are abstractly modeled as node failures independently of mutation type; both missense and loss-of-function mutations are treated equivalently, under the assumption that they affect network behavior and PPIs. This choice deliberately restricts the analysis to topological properties of the interactome, excluding mutation-specific mechanisms and regulatory or dynamical effects, which are instead explicitly modeled in other approaches based on gene regulatory networks (e.g.,^46^). In general, our research contributes to the relatively new conceptual framework of the theory of genetics and molecular biology. In fact, it has become clear that a gene alone does not have a specific function; it must instead be seen as a means that cells use for their functioning in interaction with the other parts of the genome and the environment.^47^^,^^48^ *Interaction* is the key word here, and, as we showed, the genes that are the most important for the integrity of the interactome are also crucial for keeping the cell alive. However, they cannot be the majority of the genes that are involved in cancers, because otherwise cancer cells would not be able to remain viable. In fact, genes mutated in cancer are a mixture between genes that are the most important for maintaining network resilience and genes that are marginal in the network topology (random genes).

### Limitations of the study

Genetic mutations are abstractly modeled as node failures, independently of mutation type; both missense and loss-of-function mutations are treated equivalently, under the assumption that they affect network behavior and PPIs. This choice deliberately restricts the analysis to topological properties of the interactome, excluding mutation-specific mechanisms and regulatory or dynamical effects, and it is, of course, an important approximation.

Moreover, the analyses performed in this work require a large number of mutated genes, as the applied resilience is a global network property and the measured effects are individually small. In fact, in cases resulting from only a small number of mutated genes, they cannot be observed. This is at the same time a strength and a limitation. One reason it constitutes a strength is that it allows our results to be reliable, as they are based on a large dataset. In the same vein, that strength becomes a limitation because it makes it impossible to conduct the same analyses in some other contexts, for instance, in cancers with a small number of mutations. However, even in those cases, the results shown here can be used, and the ranking applied to the specific mutated genes, with no need to perform the whole analysis.

## Resource availability

### Lead contact

Requests for further information and resources should be directed to and will be fulfilled by the lead contact, Dr. Stefano Polizzi (stefano.polizzi@unibo.it).

### Materials availability statement

This study did not generate new materials or unique reagents.

### Data and code availability

• The dataset analyzed in this study is a public database consisting of somatic gene-level mutational information of the Pan-Cancer Analysis of Whole Genomes (PCAWG).^2^
• To perform the analysis, a graph consisting of the union of different interactomes was created.^20^^,^^49^^,^^50^^,^^51^^,^^52^^,^^53^
• The analyses were generated using Python version 3.13.02.
• The code used for the analysis, the PCAWG mutation list, and the union interactome can be freely found in the GitHub repository (https://github.com/ilboia/Interactome-Resilience-of-Pan-cancer-Mutations).

## Acknowledgments

We thank Lorenzo Dall’Olio for fruitful discussions. We acknowledge support by the 10.13039/501100000780European Union—Horizon 2020/2023 program and Innovative Health Initiative, 10.13039/501100004907IHI (GenoMed4All project #101017549 to G.C.; Synthema project, #1101095530 to G.C.; Synthia project #101172872 to G.C); AIRC Foundation (Associazione Italiana per la Ricerca contro il Cancro, Milan Italy—Project #26216 to G.C; PRIN (Ministry of University & Research, Italy—Project 20229B28PE to G.C).

## Author contributions

Conceptualization, S.P., N.B., and T.M.; methodology and visualization, S.P., N.B., T.M., and M.T.; data curation and investigation, S.P., N.B., and M.T.; formal analysis, S.P. and T.M.; resources, S.P., T.M., and G.C.; supervision, S.P., T.M., and G.C.; validation, S.P., N.B., and G.C.; writing – original draft and software, S.P., N.B.; writing – review and editing, all authors; all authors have read and approved the final submitted manuscript.

## Declaration of interests

The authors declare no competing interests.

## STAR★Methods

### Key resources table

**Table:** 

REAGENT or RESOURCE	SOURCE	IDENTIFIER
**Software and alghorithms**		

Python 3.13.2	Python Software Foundation	https://www.python.org/downloads/release/python-3132/
R 4.4.1	The R Foundation	https://cran.r-project.org/bin/windows/base/old/4.4.1/
igraph	The R Foundation	https://r.igraph.org
NetworkX 3.4.2	Python Software Foundation	https://pypi.org/project/networkx/3.4.2/
GSEApy 1.1.8	Python Software Foundation	https://pypi.org/project/gseapy/1.1.8/

**Deposited data**		

Bioplex	Huttlin EL, Bruckner RJ, Paulo JA, Cannon JR, Ting L, Baltier K et al. Architecture of the human interactome defines protein communities and disease networks. Nature. 2017;545(7655):505–509.^49^	https://bioplex.hms.harvard.edu/interactions.php
Cofrac15	Wan C, Borgeson B, Phanse S, Tu F, Drew K, Clark G et al. Panorama of ancient metazoan macromolecular complexes. Nature. 2015;525(7569):339–344.^50^	https://static-content.springer.com/esm/art%3A10.1038%2Fnature14877/MediaObjects/41586_2015_BFnature14877_MOESM13_ESM.zip
HuRI	Luck K, Kim DK, Lambourne L, Spirohn K, Begg BE, Bian W et al. A reference map of the human binary protein interactome. Nature. 2020;580(7803):402–408^20^	https://interactome-atlas.org/download
Cpathdb	Herwig R, Hardt C, Lienhard M, Kamburov A. Analyzing and interpreting genome data at the network level with ConsensusPathDB. Nature protocols. 2016;11(10):1889–1907.^51^	http://cpdb.molgen.mpg.de/MCPDB
Dmnd	Ghiassian SD, Menche J, Barabási AL. A DIseAse MOdule Detection (DIAMOnD) algorithm derived from a systematic analysis of connectivity patterns of disease proteins in the human interactome. PLoS computational biology. 2015;11(4):e1004120.^52^	https://journals.plos.org/ploscompbiol/article/file?type=supplementary&id=10.1371/journal.pcbi.1004120.s003
Lit-BM	Luck K, Kim DK, Lambourne L, Spirohn K, Begg BE, Bian W et al. A reference map of the human binary protein interactome. Nature. 2020;580(7803):402–408^20^	https://www.interactome-atlas.org/data/Lit-BM.tsv
FpClass	Kotlyar M, Pastrello C, Pivetta F, Lo Sardo A, Cumbaa C, Li H et al. In silico prediction of physical protein interactions and characterization of interactome orphans. Nature methods. 2015;12(1):79–84.^53^	https://mybiosoftware.com/fpclass-interactions-and-properties-of-human-proteins.html
PCAWG	Campbell P, Getz G, Korbel J, Stuart J, Jennings J, Stein L et al. Pan-cancer analysis of whole genomes. Nature. 2020;578(7793):82–+. https://doi.org/10.1038/s41586-020-1969-6^2^	https://www.cbioportal.org/study/summary?id=pancan_pcawg_2020
TCGA	National Cancer Institute Center for Cancer Genomics	https://www.cancer.gov/ccg/research/genome-sequencing/tcga

**Other**		

Softwares for isolating the nodes and resilience calculation	This Paper	https://github.com/ilboia/Interactome-Resilience-of-Pan-cancer-Mutations

### Method details

#### Human PPI

The analysis was based on the graph union of 7 different interactomes, which are all physical protein interactions, we chose the principal high-throughput (HT) interactomes: bioplex,^54^ cofrac15,^49^ HuRI^20^; Integrative Curated (ITC): cpathdb,^50^ dmnd,^51^ Lit-BM^20^; Integrative Predictive (IP): FpClass.^52^ The graph union between them is done to be more complete and include the highest possible number of genes, while keeping the highest possible link accuracy, since HT interactomes are known to have a high rate of false negative interactions.^53^ After pruning duplicate edges and self-loops, the resulting network comprises *N* = 17462 nodes, an average degree of *d* = 68.3, a number of links of 598982, and a link density of 0.0039. To standardize gene nomenclature across datasets, we used EnsDb.Hsapiens.v86.^55^ This analysis was performed in R 4.4.1 using the igraph package.^56^

#### Interactome resilience

In the context of biological networks, several resilience measures exist. While some of them focus on global resilience by subsequently isolating random nodes,^22^ others try to assess the impact of specific perturbations on the network information flow^57^ and others are local measures computing how much a single node removal changes the size of the largest connected component.^58^ We studied the resilience of the interactome networks under successive node isolation using a resilience of the first type,^22^ which quantifies the level of network fragmentation when an increasing fraction *f* of its nodes is removed. Fixed *f*, network fragmentation was quantified by the modified Shannon diversity, which can be interpreted as the entropy of the isolated components of the graph:

(Equation 1) $$S\left(f\right)=-\frac{1}{\log\left(N\right)}\sum_{i=1}^{c}\left[p_{i}\;\log\left(p_{i}\right)\right]$$

where *N* is the number of nodes of the interactome, *c* is the number of disconnected components, and *p*_*i*_ their relative size, that is, *p*_*i*_ = *n*_*i*_/*N* with *n*_*i*_ the number of nodes of component *i*. The overall network resilience is defined as:

(Equation 2) $$R=1-\int_{0}^{1}S\left(f\right)df$$

and takes values in [0,1]. The higher *R*, the more resilient to mutations is the network. This resilience measure is adapted to pan-cancer studies because, being a global measure, it needs a large number of mutated genes (more than at least 40% of the genes of the interactome) to be effective.

We studied the overall resilience of the reconstructions of the human interactome under two different failure schemes.

• *Random Failures*: nodes (genes/proteins) whose interactions are removed were randomly chosen with uniform distribution. This simulates the real genetic mutations normally occurring in living cells.
• *Targeted Failures*: nodes (genes/proteins) were preferably chosen with a specific order. For instance, they were selected in decreasing order of node degree and clustering coefficient.

The same failure schemes were also tested, removing the considered node and its first neighbors at each simulation step, but they will not be part of the main discussion of this paper. Note that we will interchangeably use the terms *node removal* or *node failure* to mean this procedure of removing all links of a node from the network, creating an isolated component.

Figure 1 reports an illustrative example of the link between network fragmentation, *S* and *R* (for details on the definition of *S* and *R* see Methods S1). It also provides a schematic view of the two removal schemes.

#### Standard networks

We tested the resilience measure on several standard networks, each obtained with parameters such as having the same link density of the interactome and the same number of nodes. For the scale-free network parameter, we selected the value that provided the best fit to the degree distribution of the interactome. The number of nodes was kept identical to that of the interactome, except in the case of the fully connected network, where it was reduced by half due to memory constraints. *Random Regular. d* = 68; *Fully Connected. N* = 17462/2; *Erdős-Rényi. p* = 0.039 (link probability); *Scale Free. m* = 6 (number of new links to existing nodes). For each random network, simulations were performed on 10 independently sampled networks, with statistics computed over 100 repetitions for each (1000 repetitions total). In cases where the removal order was deterministic (e.g., targeted removal in random regular networks), variability was assessed solely based on network generation. This analysis and all resilience simulations were performed in Python 3.13.2.

#### Mutation dataset

From the cbioportal platform (https://www.cbioportal.org/datasets, accessed in June 2024) we collected somatic gene-level mutational information of the Pan-Cancer Analysis of Whole Genomes (PCAWG) database, including 38 different tumor types for 2658 patients,^2^ in order to measure the effect on the resilience of the whole cancer pathology. The dataset includes mutations as single-nucleotide variants (SNVs) and small insertions/deletions (indels). A gene was considered mutated in a given sample if at least one somatic mutation affecting that gene was present. The number of reported genes is 17335, of which 15800 overlap with the genes of the consensus interactome. The PCAWG is a recent and reliable dataset integrated under a uniform processing pipeline to ensure consistency across tumor types, allowing for a robust pan-cancer analysis.^2^ We did not perform any posterior operations on the public data.

All our analyzes were also validated on the TCGA pancancer cohort (https://tcga-data.nci.nih.gov, accessed in June 2024), resulting from the Pan-Cancer Atlas project, containing cancer projects collected between 2013 and 2018.^59^^,^^60^ Although this dataset was less robust for pan-cancer comparison, it served as a valuable resource to corroborate the results.

#### Functional enrichment analysis

We used the database gene ontology (GO) to perform functional enrichment analysis, in order to study what biological functions are significantly over-represented in the selected set of genes.^29^^,^^30^ Biological processes enrichment was performed using the ENRICHR module from the GSEApy python library^61^ and the Gene Ontology (GO) “GO Biological Process 2025″ gene set for the human organism.^62^ Since 15800 genes are shared between the PCAWG mutation dataset and the selected interactome, we considered the whole human genome to be an appropriate background gene set for the enrichment analysis. The *p*-values of the test on the processes enrichment of the selected genes are adjusted with the Benjamini–Hochberg correction.

Functional enrichment analysis was performed with over-representation analysis (ORA) using the Bioconductor packages “ClusterProfiler”,^63^ “g:profiler”.^64^ Representation of enriched over and under expressed processes was performed using EnrichmentMap and Annotables on Cytoscape.^65^

### Quantification and statistical analysis

To assess the resilience of the networks using the algorithms that involve random nodes selection, we performed multiple runs of the removal algorithms, and where appropriate multiple runs on different randomly generated networks (the number of repetitions is specified where necessary). Resilience distributions were tested to be normally distributed and their mean values and associated standard errors of the means were given as final measures.

That allowed us to apply the *t* test to all the measures we performed on the networks’ resilience.

When evaluating the functional enrichment, the GSEApy Enrichr function computed the over-representation analysis applying the Fisher’s exact test, and *p*-values were adjusted for multiple hypothesis testing using the Benjamini-Hochberg (BH) correction. A significance threshold of adjusted *p* < 0.05 was applied.

### Additional resources

The remotion algorithm was implemented using Python 3.13.2, NetworkX and other python libraries. Detailed information about the library versions and the implemented algorithms could be found in the GitHub repository (https://github.com/ilboia/Interactome-Resilience-of-Pan-cancer-Mutations).

## Supplemental information

Document S1. Figures S1−S6 and Table S1

Table S2. High-impact genes identification – Full list, related to Section Results- Interactome Resilience to pan-cancer mutations In the following table, we show all the genes that are identified as high-impact for interactome resilience, in decreasing order from the greatest impact. That list is the result of the algorithm that removes all the genes in decreasing mutation order except for the selected one, which will be removed last (together with nodes with 0 mutations). Those are the genes that cause the resilience to increase the most when considered as non-mutated.

Table S3. Significantly enriched biological processes for high-impact genes on interactome resilience, related to Section Results - Functional enrichment of vulnerable nodes In this table are listed all the significantly enriched biological processes (adjusted p value >0.05) of the genes that cause the greatest change in resilience. See Table S2 for the genes’ list.
