# Supplementary material for: Network resilience of the human interactome to pan-cancer mutations reveals conserved pathway vulnerabilities
Source: iScience. 2026 Mar 27;29(5):115500. doi: 10.1016/j.isci.2026.115500 (PMC13091781; doi:10.1016/j.isci.2026.115500)
Supplement: Document S1. Figures S1−S6 and Table S1 [file mmc1.pdf]

iScience, Volume *29*

Supplemental information

Network resilience of the human interactome to pan-cancer mutations reveals conserved pathway vulnerabilities

Stefano Polizzi, Nicolas Biondini, Tommaso Matteuzzi, Martina Tarozzi, and Gastone Castellani

Methods S1: Global Network Resilience, related to Section *STAR Methods - Interactome resilience*

Global Network Resilience was defined following [1]. Let's call I a network of N nodes. If we remove a fraction f of nodes from I, the network is fragmented in a set of c components of different sizes. Let's call s_i, with $i = 1, \ldots, c$, the number of nodes in the i-th component.

The *Shannon Diversity* for the resulting components set is defined as:

$$S(I_f) = -\frac{1}{log(N)} \sum_{i}^{c} p_i log p_i \tag{1}$$

where $p_i = \frac{s_i}{N}$ and I_f denotes the set of componets originating from I when a fraction of nodes f is removed. The factor $\frac{1}{log(N)}$ is introduced to allows the comparison of interactomes of different size [1].

In this definition, each removed node is a component of size $s = 1$ and the corresponding p is $p_{(1)} = 1/N$, so that the contribution to S of the $n = Nf$ removed nodes is given by:

$$S = N \left[-\frac{1}{log(N)} \cdot \frac{1}{N} log \left(\frac{1}{N} \right) \right] f = f \tag{2}$$

Maximum Resilience

For a network I of N nodes, after removal of $n = Nf$ nodes, the minimum possible Shannon Diversity, S, is obtained when only a giant component of dimension $(N - n)$ is present, in this case, the sum in (1) has only two terms and, injecting Eq (2), $S(I_f)$ reads:

$$S(I^1_{f=\frac{n}{N}}) = \frac{n}{N} - \frac{1}{log(N)} \left[\frac{N-n}{N} log \left(\frac{N-n}{N} \right) \right] \tag{3}$$

Substituting $n = Nf$ we have:

$$S(I^1) = f - \frac{1}{log(N)} (1-f) log(1-f) \tag{4}$$

This corresponds to the maximum possible resilience, which for $N \to \infty$ is the bisectrix $S(I^1) = f$.

Figure S1: Interactome degree distribution, related to Section *Results - Comparison of interactome resilience with standard networks*

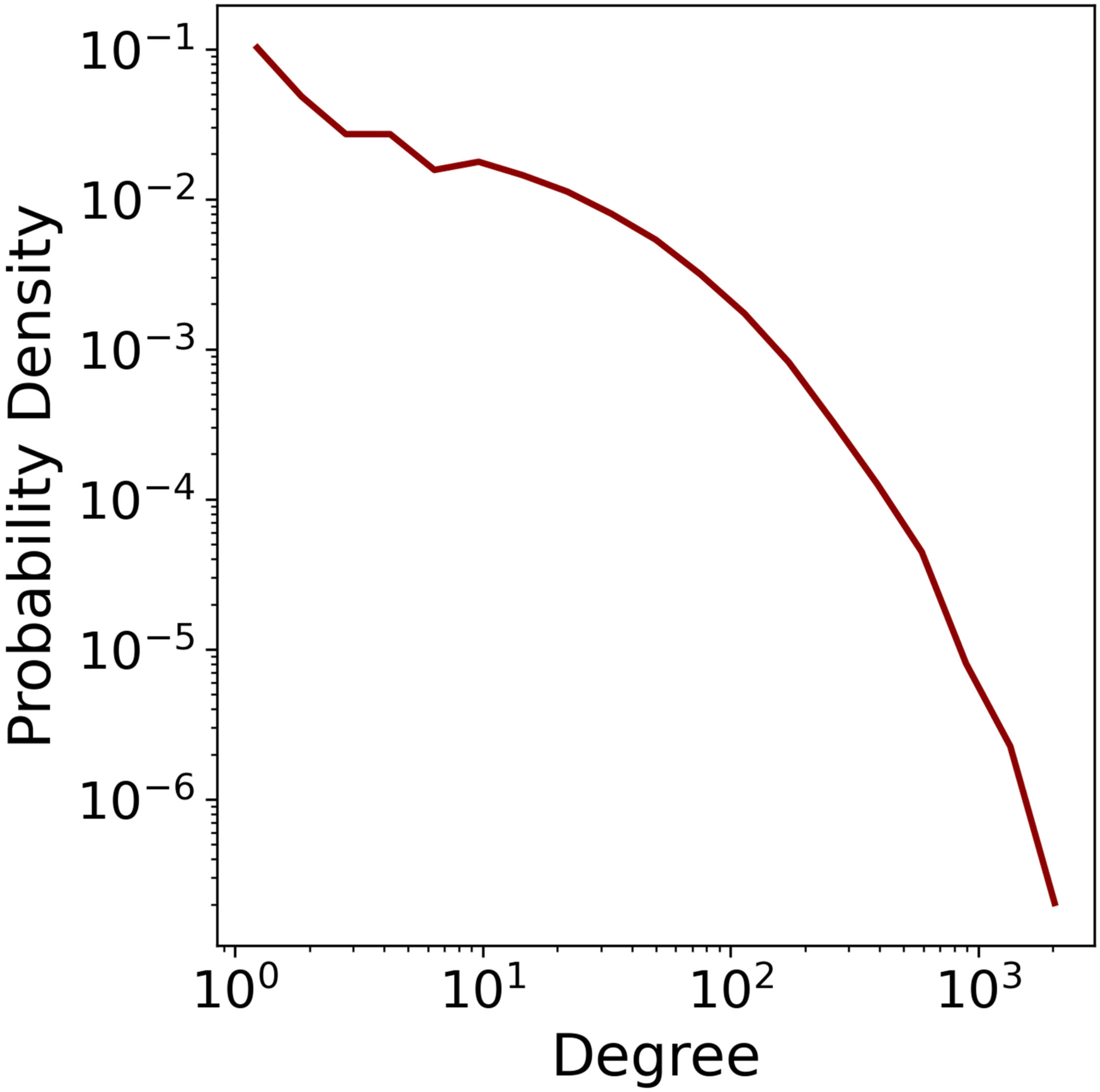

Fig 1. Degree distribution of the consensus interactome.

Figure S2: Resilience of PPI network for the TCGA dataset, related to Section *Results - Interactome Resilience to pan-cancer mutations*

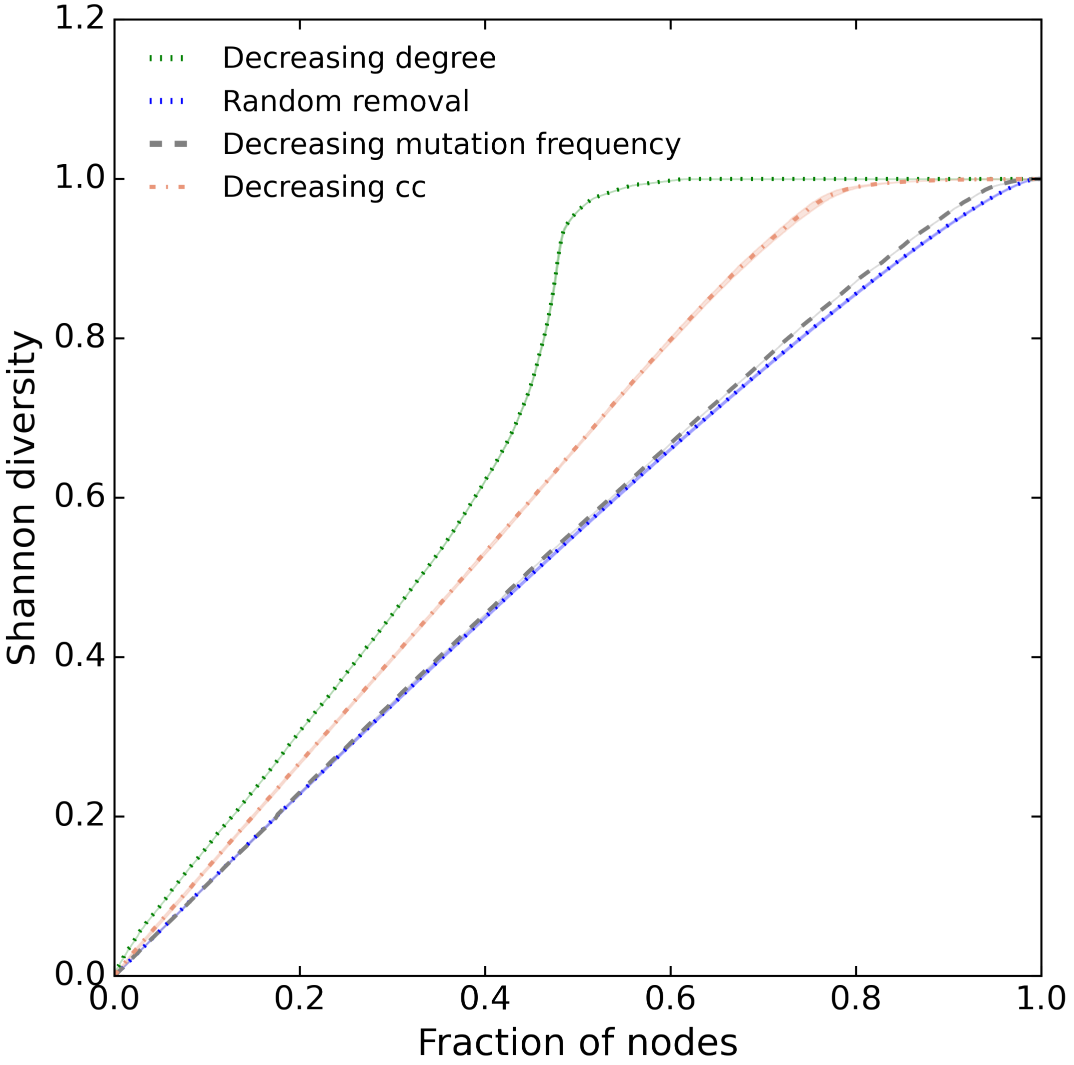

Fig 2. Shannon diversity curves of PPI network for the TCGA dataset.

Figure S3: Shannon diversity curves when removing nodes of the same degree as the mutated ones but chosen randomly, related to Section *Results - Interactome Resilience to pan-cancer mutations*

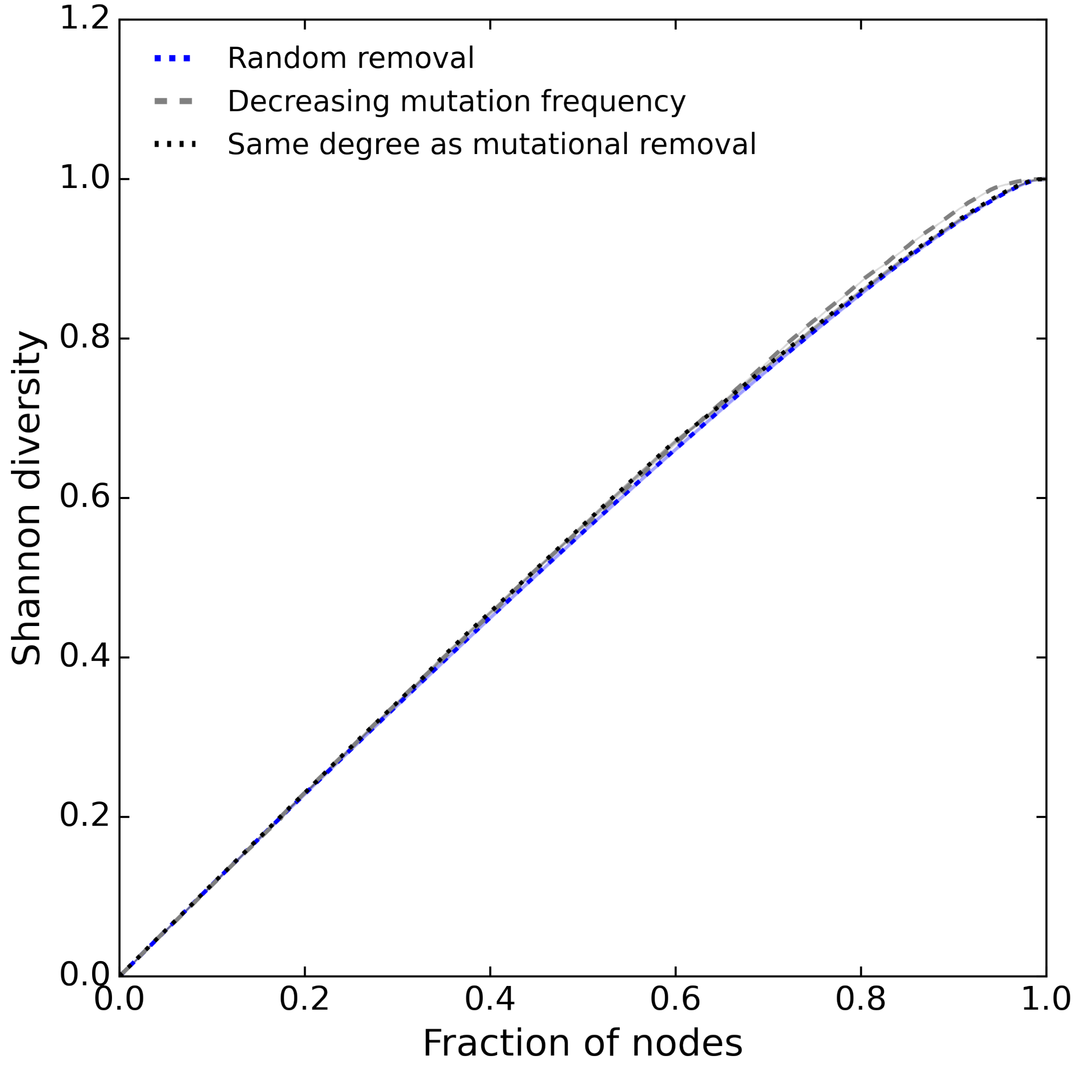

Fig 3. Shannon diversity curves when removing nodes of the same degree as the mutated ones but chosen randomly, instead of being removed in order of frequency of mutation.

Figure S4: Single node impact on the resilience when removing nodes in decreasing degree order, related to Section *Results - Interactome Resilience to pan-cancer mutations*

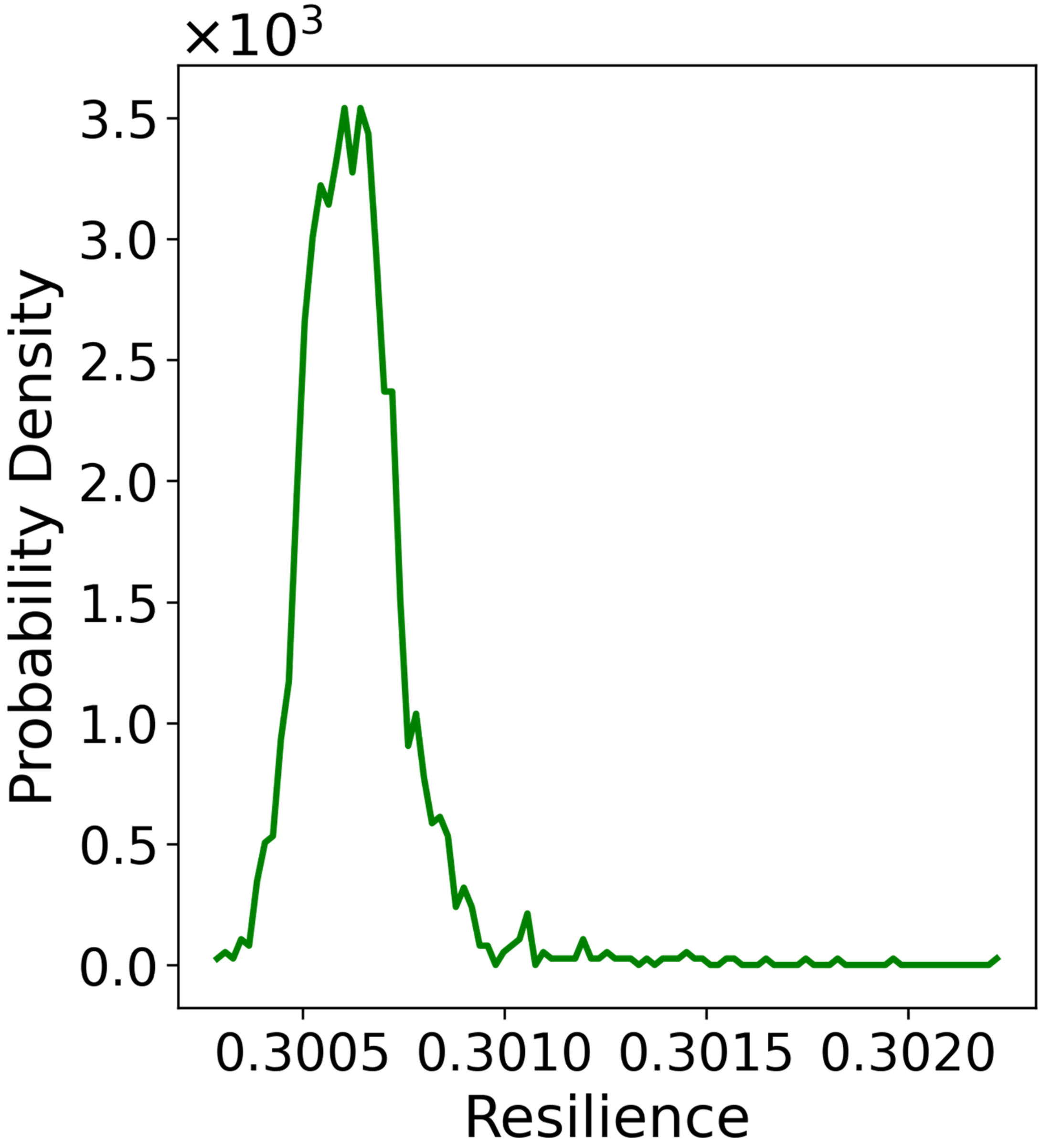

Fig 4. Probability density of resilience values due to the impact of a single gene with targeted removal in decreasing degree order. In turn each randomly selected gene is removed at the end.

Figure S5: Resilience of the interactome removing a node and all its first neighbors, related to Section *Results - Interactome Resilience to pan-cancer mutations*

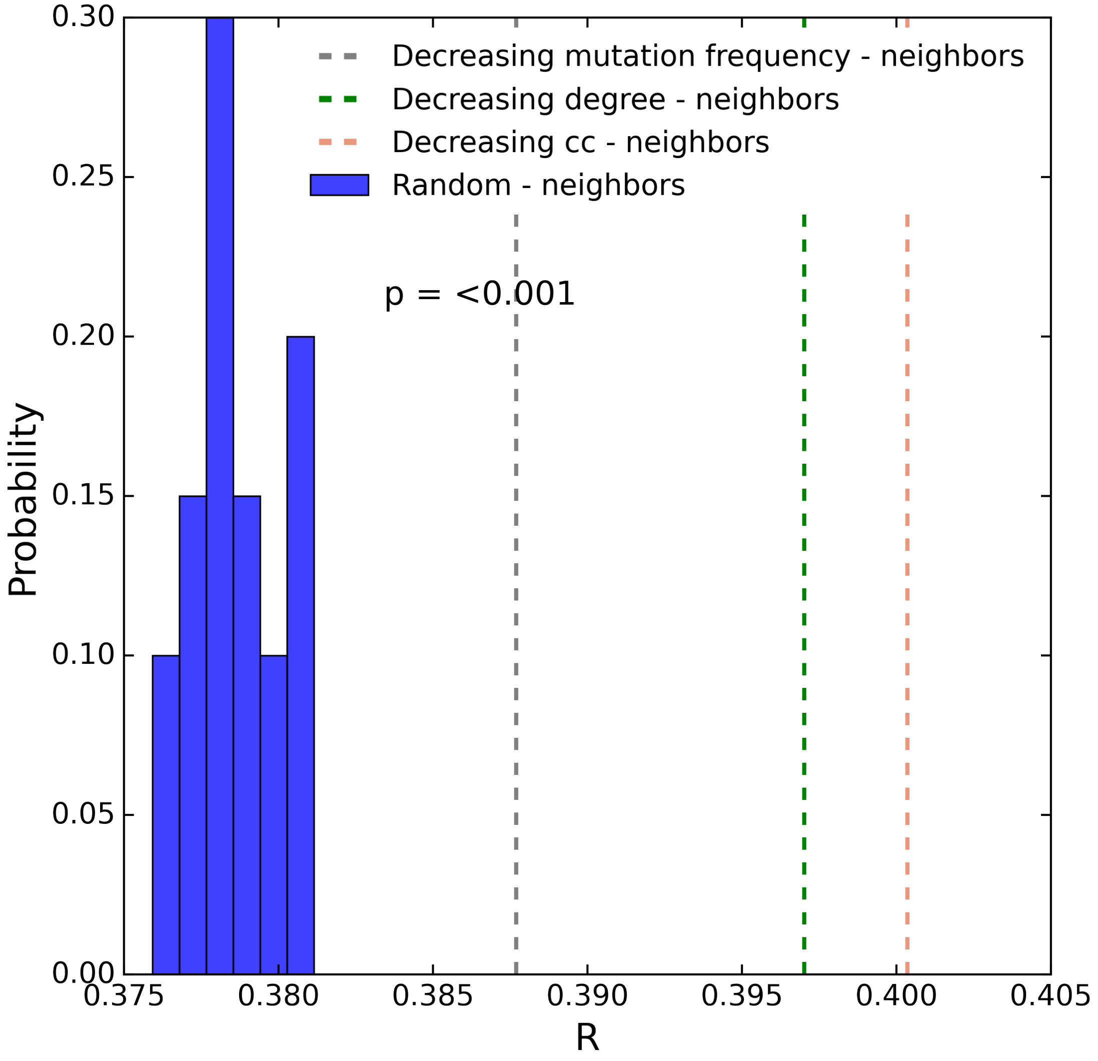

Fig 5. Distribution of resilience values for random removal and value of resilience for decreasing mutation frequency (dotted grey line), decreasing degree (dotted green line) and decreasing clustering coefficient (dotted orange line), when removing the central node and all its first neighbors.

Figure S6: Comparison of clustering coefficient distributions, related to Section *Results - Interactome Resilience to pan-cancer mutations*

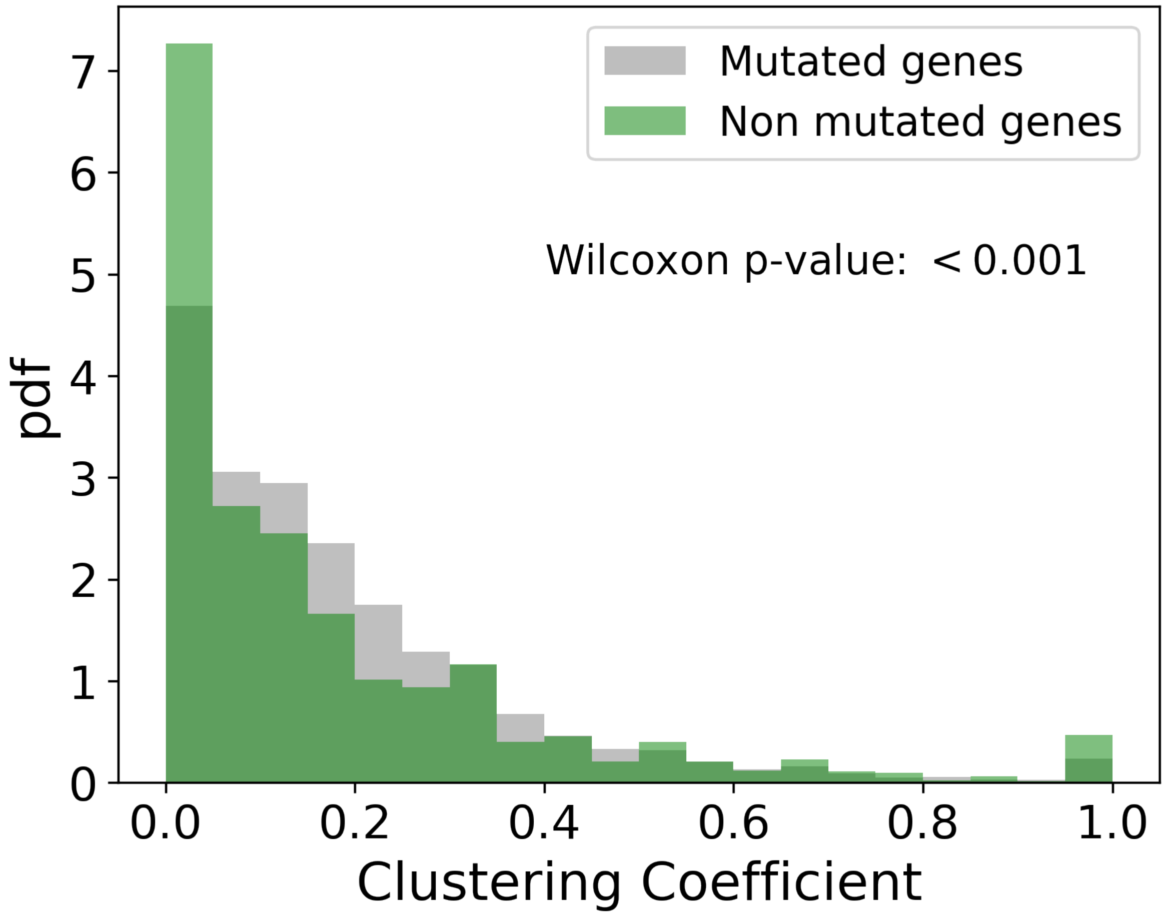

Fig 6. Comparison of the clustering coefficient distributions for mutated genes vs non-mutated genes, and p-value of the Wilcoxon-Mann-Whitney test.

Table S1: Neighbors-removal resilience, related to Section *Results - Interactome Resilience to pan-cancer mutations*

Table 1. In the following table we print the resilience of the PPI when removing each node and all of its first neighbors. When a node is selected also all its neighbors are removed and only when all the neighbors are removed the following node is chosen.

Removal type	Resilience	Standard Deviation
Decreasing mutations + neighbors	0.388	-
Decreasing degree + neighbors	0.397	1.46×10^{-4}
Random + neighbors	0.379	1.52×10^{-3}
Decreasing CC + neighbors	0.400	3.3×10^{-5}

References

[1] Marinka Zitnik et al. “Evolution of resilience in protein interactomes across the tree of life”. In: *Proceedings of the National Academy of Sciences* 116.10 (2019), pp. 4426–4433.
